# Dynamic Portfolio Strategy Using Clustering Approach

**DOI:** 10.1371/journal.pone.0169299

**Published:** 2017-01-27

**Authors:** Fei Ren, Ya-Nan Lu, Sai-Ping Li, Xiong-Fei Jiang, Li-Xin Zhong, Tian Qiu

**Affiliations:** 1 School of Business, East China University of Science and Technology, Shanghai 200237, China; 2 Research Center for Econophysics, East China University of Science and Technology, Shanghai 200237, China; 3 Institute of Physics, Academia Sinica, Taipei 115 Taiwan; 4 College of Information Engineering, Ningbo Dahongying University, Ningbo 315175, China; 5 School of Finance, Zhejiang University of Finance and Economics, Hangzhou 310018, China; 6 School of Information Engineering, Nanchang Hangkong University, Nanchang 330063, China; Universidad Veracruzana, MEXICO

## Abstract

The problem of portfolio optimization is one of the most important issues in asset management. We here propose a new dynamic portfolio strategy based on the time-varying structures of MST networks in Chinese stock markets, where the market condition is further considered when using the optimal portfolios for investment. A portfolio strategy comprises two stages: First, select the portfolios by choosing central and peripheral stocks in the selection horizon using five topological parameters, namely degree, betweenness centrality, distance on degree criterion, distance on correlation criterion and distance on distance criterion. Second, use the portfolios for investment in the investment horizon. The optimal portfolio is chosen by comparing central and peripheral portfolios under different combinations of market conditions in the selection and investment horizons. Market conditions in our paper are identified by the ratios of the number of trading days with rising index to the total number of trading days, or the sum of the amplitudes of the trading days with rising index to the sum of the amplitudes of the total trading days. We find that central portfolios outperform peripheral portfolios when the market is under a drawup condition, or when the market is stable or drawup in the selection horizon and is under a stable condition in the investment horizon. We also find that peripheral portfolios gain more than central portfolios when the market is stable in the selection horizon and is drawdown in the investment horizon. Empirical tests are carried out based on the optimal portfolio strategy. Among all possible optimal portfolio strategies based on different parameters to select portfolios and different criteria to identify market conditions, 65% of our optimal portfolio strategies outperform the random strategy for the Shanghai A-Share market while the proportion is 70% for the Shenzhen A-Share market.

## Introduction

Portfolio management is one of the hottest issues in finance. It primarily concerns with the best combination of securities for specific profits that investors need. The fundamental theory about portfolio optimization can be traced back to the Markowitz framework [1], which selects the allocation of investors’ investment based on a mean-variance analysis. A great deal of work has subsequently contributed to the study of portfolios by using a variety of alternative methods, e.g., neural networks [2–4], genetic algorithms [5, 6], simulated annealing [7], Random Matrix Theory (RMT) filtering [8, 9], and hierarchical clustering [10–13]. Among them, hierarchical clustering is one of the most efficient methods for the selection of a basket of stocks for optimal portfolios. In fact, the selection of a set of stocks is a pre-requisite for the Markowitz theory [14, 15], since it is dedicated to the investment proportion of a limited number of selected stocks.

By using the hierarchical clustering method, the correlations between shares are revealed by the topological structure of the constructed stock network [16–25], and can be further applied to portfolio optimization. A minimum spanning tree (MST) [26] description of the correlations between stocks has shown that the stocks included in the minimum risk portfolio (the optimal Markowitz portfolio) tend to lie on the outskirts of the asset network [10, 27]. By extracting the dependency structure of financial equities using both MST and planar maximally filtered graphs (PMFG) methods, it has been found that the portfolios from a selection of peripheral stocks have lower risk and better returns than the portfolios from a selection of central stocks, the centrality/peripherality of which are measured by indices such as degree, betweenness centrality, eccentricity, closeness and eigenvector centrality [11]. A K-means clustering algorithm and its extension C-means clustering algorithm are applied to the classification of stocks. The stocks selected from these classified groups are used for building portfolios, which perform better than the benchmark index [12]. Similar works have been done in India, Taiwan and China stock markets using the same K-means cluster analysis [13, 14]. Furthermore, clusters or communities detected from network graphs also provide useful information for correlations among stocks [19, 22, 28, 29], which has also been used in the stock selection of portfolios [30–32]. This type of method is analogous to the clustering algorithm for their similarities in stock selections from clusters or communities partitioned by specific approaches.

Evidence from recent studies suggests that the topological structures of stock networks are evolving over time and changes markedly during financial crises [22, 28, 29, 33–38]. Therefore, a fixed set of stocks is not a wise choice for the portfolio selection under different market conditions. One possible way of solving this problem is to identify the stock clusters based on the network graphs in different time periods (moving windows) and perform the portfolio selection from the identified clusters in each period [11]. An alternative way is to use the dynamic conditional correlations (DCC) method to estimate time-varying correlations among stock returns based on the Markowitz framework [39–41]. A new estimator called detrended cross-correlation coefficients (DCCA) is also used to describe the correlations between nonlinear dynamic series [42–45]. Other works refer to evolutionary algorithms include [14, 46].

The main motivation of this paper is to propose a new dynamic portfolio strategy based on the time-varying structures of the financial filtered networks in the Chinese stock markets. A moving window with size *δt* is used to study the variance of stock networks over time *t*. We choose the MST method to filter out the network graph in each window for its validity and simplicity, which is generated by connecting the nodes with the most important correlations. The portfolio selection is determined by the network structure in the previous window (selection horizon), which is picked from a selection of peripheral stocks, most diverse corresponding to the Markowitz portfolio with minimum variance [11], and central stocks, highly correlated and synchronous in price movements. The selected portfolios are subsequently used for investment in the following investment horizon.

The underlying market conditions are further considered in our dynamic portfolio strategy, which comprises the investment strategy together with the portfolio selection. A recent study has verified that accurate price and volatility predictions can be used as a basis for the particular trading strategy adopted for the portfolio [31]. In our study here, we presume that the optimal portfolio can change under different market conditions, and portfolio investments are implemented based on both historical price changes and price predictions in the future. For simplicity, three market conditions: drawup and drawdown trends of the daily price and a relatively stable status in between will be used in the selection and investment horizons. A variety of selected portfolios will be compared under different combinations of market conditions in the two horizons, and the optimal portfolio with the largest profit will be identified under each market condition. To further testify the efficiency of our dynamic portfolio strategy, we will carry out a training process using the first half of the sample data, and use the optimal portfolio obtained from training to make investments using the remaining half of the sample data. As will be shown below, our optimal portfolios outperform the benchmark stock index on average.

## Data and Methods

Our daily data include 181 × 2 stocks listed on Shanghai and Shenzhen A-Share markets, which have the largest volumes in two major stock exchanges in mainland China, over the period of 15 years from January 1, 2000 to December 31, 2014. To ensure the continuity and integrity of the data, the stocks selected in our study are most actively traded stocks throughout the sample period. For this purpose, we filter out those stocks which were once suspended from the market for more than 46 trading days, about 1% of a total of 3,627 trading days. This filtering process yields the sample data including 181 A-Share stocks and 643,404 and 639,607 daily records in total for the two markets respectively. The return series of a certain stock *i* is computed as *r*_*i*_(*t*) = ln *P*_*i*_(*t*) − ln *P*_*i*_(*t* − 1), where *P*_*i*_(*t*) is the closing price for stock *i* on the *t*-th day. The price returns for 181 × 2 stocks are calculated, and the effects of corporate actions are eliminated, for instance the cash dividend, the bonus share, and the rights issue.

On a certain day *t*, a correlation matrix is calculated by using the Pearson correlation coefficient estimator on the returns series in the window {*t* − *δt* + 1, …, *t*}, and the stock network constructed by the MST method, see details in network construction in Methods. Ten categories of portfolios are selected respectively from a set of 10% of most peripheral and central stocks in the MST graph, the centrality/peripherality of which are measured by five parameters capturing network topology: degree, betweenness centrality, distance on degree criterion, distance on correlation criterion and distance on distance criterion. For more details, see portfolio selection in Methods. The selected portfolios are used for investment in the following horizon {*t* + 1, …, *t* + Δ*t*}, with an equal weight for each selected stock following [47], in which 1/N portfolio strategy is proven to be more efficient than the mean-variance model. The investment returns of chosen portfolios are calculated under nine combinations of market conditions in the selection and investment horizons. The market conditions include drawup (U), drawdown (D) and stable (S) status identified by trading day criterion, amplitude criterion, “OR” criterion, and “AND” criterion. Please refer to the descriptions on the identification of market conditions in Methods. Since the results obtained from the three criteria are quantitatively similar, we will only present our results based on the trading day criterion here. One then moves to *t* + *φ*. The same portfolio strategy is adopted by selecting the portfolio in the window (horizon) {*t* + *φ* − *δt* + 1, …, *t* + *φ*}, and then using the selected portfolio for investment in the horizon {*t* + *φ* + 1, …, *t* + *φ* + Δ*t*}. The investment returns of 10 categories of selected portfolios are calculated under nine combinations of market conditions in the two horizons, and the optimal portfolio is obtained by evaluating their average performances for different moving windows.

A suitable choice of *δt* and *φ* will indeed help the network capture the information of the original data as much as possible. The larger *δt* is and the smaller *φ* is, the more stable the network structure is, and the more the market information is filtered out. On the contrary, the network structure is more volatile and unauthentic [48, 49], though the temporal fluctuations can be easily noticed. Many studies have revealed that in order to ensure stocks to have enough number of trading days to be statistically significant, *δt* should be larger than the number of sample stocks *N* = 181 [50, 51]. By careful observations and precise calculations, we choose *δt* = 10 months (≈ 200 days) and *φ* = 1 month(≈ 20 days), thus having 161 daily points to be used for portfolio investments in total. The determination of the optimal values of the parameter *φ* and the size of investment horizon Δ*t* will be described in detail in network construction and determination of investment horizon in Methods respectively.

### Network construction based on MST method

Denote *r*_*i*_(*t*) and *r*_*j*_(*t*) as the logarithmic returns of stocks *i* and *j*, the Pearson correlation coefficient between their return series is given by

$$\begin{array}{c} \rho \left(i,j\right)=\frac{E\left[r_{i}r_{j}\right]-E\left[r_{i}\right]E\left[r_{j}\right]}{\sqrt{\left(E\left[r_{i}^{2}\right]-E{\left[r_{i}\right]}^{2}\right)\left(E\left[r_{j}^{2}\right]-E{\left[r_{j}\right]}^{2}\right)}}, \end{array}$$ (1)

where *E*[⋅] denotes the mathematical expectation of the sequence over time *t*. Before the construction of the MST graph, the correlation coefficient is converted into the distance between stocks *i* and *j* by the following equation

$$\begin{array}{c} d\left(i,j\right)=\sqrt{2(1-\rho (i,j))}, \end{array}$$ (2)

The distance *d*(*i*, *j*) ranges from 0 to 2, and a small distance corresponds to a large correlation coefficient. For the 181 sample stock datasets in the Shanghai and Shenzhen A-Share markets, a distance matrix with 181 × 181 elements is obtained for each market respectively. The estimation of the correlation matrix has unavoidably associated with a statistical uncertainty, due to the finite length of the return series as well as noise.

We choose the MST method to filter out the network graph in each window so as to eliminate the redundancy and noise while maintaining significant links. By constructing the minimum spanning tree, we effectively reduce the information space from *n*(*n* − 1)/2 correlation coefficients to *n* − 1 tree edges. In other words, the amount of information is being compressed dramatically. The procedure to build the MST network can be carried out as follows: First, arrange the distances between all pairs of stocks in an ascending order. Second, start by matching the nearest nodes. Continue matching according to the ordered list if and only if the graph obtained after the matching is still a tree. Edges maximizing the sum of the correlations over the connections in the tree are more likely to stay by this method. Research works such as references [16, 17, 52–54] have used the MST model to filter networks. Given the data used in our study, we choose Prim algorithm to build our network.

There exists a close relationship between correlation coefficient matrices and MST distance matrices. To investigate their relationship, we calculate the Pearson linear correlation coefficients between the value of the mean, variance, skewness and kurtosis of the elements in both matrices. Generally speaking, the mean value of the elements in the two matrices are anti-correlated, and there exist similar features for skewness. Variance, together with kurtosis of the elements in the two matrices are positively correlated. These characteristics can be expected in view of how distances are constructed from the correlation coefficients. To confirm this, we provide the Pearson linear correlation coefficients of these variables in Table 1. We find that all variables of the elements in the two matrices are strongly correlated except for kurtosis, showing that our MST network contains most of the market information. In general, with a suitable choice of *φ*, the relationship between correlation coefficient matrices and MST distance matrices will be enhanced. From a series of tests, we find that *φ* = 1 month turns out to be the optimal choice.

**Table 1:** Relationship between correlation coefficient matrices and MST distance matrices.

	mean	variance	skewness	kurtosis
Shanghai A-Share market	-0.9849	0.7971	-0.7302	0.2813
Shenzhen A-Share market	-0.9847	0.8011	-0.8624	0.2584

Pearson linear correlation coefficients between four pairs of variables. The four variables include the value of mean, variance, skewness and kurtosis of elements in correlation coefficient matrices and distance matrices for the two markets.

### Portfolio selection based on topological parameters

Five parameters are used to measure the centrality and peripherality of nodes in portfolio selection: (I) degree, (II) betweenness centrality, (III) distance on degree criterion, (IV) distance on correlation criterion and (V) distance on distance criterion. We here present a brief introduction of these parameters.

(I) Degree *K*, the number of neighbor nodes connected to a node. The larger the *K* is, the more the edges that are associated with this node.

(II) Betweenness centrality *C*, reflecting the contribution of a node to the connectivity of the network. Denote *V* as the set of nodes in the network. For nodes *i* and *j*, *C* of a node *k* can be calculated as

$$\begin{array}{c} C=\sum_{i,j\in V}\frac{\sigma_{ij}\left(V\right)}{\sigma_{ij}}, \end{array}$$ (3)

where *σ*_*ij*_ is the number of shortest routes from node *i* to node *j*, *σ*_*ij*_(*V*) is a subsector of *σ*_*ij*_ whose routes pass through this node *k*.

Distance refers to the shortest length from a node to the central node of the network. Here, three types of definitions of a central node are introduced to reduce the error caused by a single method. Therefore three types of distances are described here.

(III) Distance on degree criterion *D*_*degree*_, a central node is the node that has the largest degree.

(IV) Distance on correlation criterion *D*_*correlation*_, a central node is the node with the highest value of the sum of correlation coefficients with its neighbors;

(V) Distance on distance criterion *D*_*distance*_, a central node is the node that gives the smallest value for the mean distance.

We use the parameters defined above to select the portfolios. Nodes with the largest 10% of degree or betweenness centrality are chosen to be in the central portfolio, and nodes whose degree equals to 1 or betweenness centrality equals to 0 are chosen to be in the peripheral portfolio. Similarly, we define the nodes rank in the top 10% by distance as the stocks of the peripheral portfolios, and the bottom 10% as the stocks of the central portfolios. The difference in the definitions results from a simple reason: In an MST network, the number of peripheral nodes (i.e., leaf nodes of a network) whose degree equals to 1 and betweenness centrality equals to 0, is much larger than 10% of the total nodes. We need to mention that it makes no difference to our results if we select randomly from these peripheral nodes so as to equal to the number in each portfolio.

The central portfolios and the peripheral portfolios represent two opposite sides of correlation and agglomeration. Generally speaking, central stocks play a vital role in the market and impose strong influence on other stocks, whereas the correlations between peripheral stocks are weak and contain more noise than central stocks. We have learned in our study that the two kinds of portfolios have their own features under different market conditions.

### Determination of investment horizon

In this subsection, we will discuss the optimal choice of the length of investment horizons Δ*t*. In general, the length of the investment horizons cannot be too long, otherwise the topological properties of the network will change and the selected central or peripheral portfolios will change accordingly. On the other hand, the length of the investment horizons cannot be too short, or the returns will be greatly influenced by market noises or exogenous events. Here, we compare the profits gained in different time horizons, namely 1 month, 5 months, 10 months and 15 months.

Sharpe ratio, defined as the ratio of the expected value of the excess returns to its standard deviation [55], is widely used to evaluate the performance of portfolios in practice [56, 57]. Table 2 shows Sharpe ratios of portfolios with different investment horizons, in which there is no particular length that could maximize Sharpe ratio in all circumstances. What is more, a one-way ANOVA is used to test the equality of excess returns gained in different lengths of investment horizons. In Table 2, the *p*-values for different horizons are all insignificant, which indicates that the choice of the horizon length does not affect the portfolio returns. In order to match the length of selection horizons and investment horizons, and to facilitate our identification of the market conditions, we choose Δ*t* = 10 months for portfolio investments.

**Table 2:** Comparison between excess returns for different lengths of investment horizons.

		Shanghai A-Share market					Shenzhen A-Share market				
		1 month	5 month	10 month	15 month	*p*-value	1 month	5 month	10 month	15 month	*p*-value
*K*	peripheral	-0.0113	0.0024	-0.0005	-0.0023	0.9968	-0.0084	0.0025	-0.0007	-0.0028	0.9964
	central	0.0455	0.0112	0.0043	0.0002	0.9652	0.0444	0.0120	0.0061	0.0030	0.9892
*C*	peripheral	-0.0113	0.0024	-0.0005	-0.0023	0.9968	-0.0084	0.0025	-0.0007	-0.0028	0.9964
	central	0.0387	0.0058	0.0016	-0.0015	0.9635	0.0281	0.0074	0.0028	0.0002	0.9915
*D*_*degree*_	peripheral	-0.0379	-0.0488	-0.0724	-0.1228	0.9978	-0.0281	-0.0048	-0.0054	-0.0072	0.9954
	central	0.0349	0.0386	0.0446	0.0367	0.9941	0.0389	0.0155	0.0069	0.0033	0.9923
*D*_*correlation*_	peripheral	-0.0317	-0.0109	-0.0079	-0.0084	0.9991	-0.0385	-0.0043	-0.0052	-0.0067	0.9911
	central	0.0291	0.0061	0.0041	0.0016	0.9958	0.0450	0.0121	0.0067	0.0033	0.9912
*D*_*distance*_	peripheral	-0.0351	-0.0091	-0.0067	-0.0079	0.9979	-0.0356	-0.0024	-0.0048	-0.0068	0.9876
	centeral	0.0195	0.0075	0.0038	0.0010	0.9984	0.0268	0.0107	0.0052	0.0020	0.9966

Sharpe ratio of portfolios for different lengths of investment horizons and ANOVA test of portfolios’ excess returns. Portfolios listed include central and peripheral portfolios selected with respect to *K*, *C*, *D*_*degree*_, *D*_*correlation*_ and *D*_*distance*_ for the Shanghai and Shenzhen A-Share markets.

### Identification of market conditions

Market conditions, which describe the general trend of the market index over a specific horizon, are measured by using four criteria: (I) trading day criterion, (II) amplitude criterion, (III) “OR” criterion, and (IV) “AND” criterion. The market indices corresponding to the Shanghai A-Share market and the Shenzhen A-Share market are the Shanghai A-Share Index and the Shenzhen A-Share Index respectively.

(I) Trading day criterion. The ratio *r*_*d*_ of the number of days with rising index to the total number of trading days in a specific time window is given by

$$\begin{array}{c} r_{d}=\frac{{N_{i}}^{+}}{N_{i}}, \end{array}$$ (4)

where ${N_{i}}^{+}$ is the number of days in which the closing price is larger than that of the previous day and *N*_*i*_ is the total number of trading days in the *i*-th time window. The ratio *r*_*d*_ ranges from 0 to 1. A large value of *r*_*d*_ represents a drawup condition while a small value of *r*_*d*_ represents a drawndown condition. With the thresholds *θ*_+_ and *θ*_−_, we identify a drawup condition if *r*_*d*_ > *θ*_+_, a drawdown condition if *r*_*d*_ < *θ*_−_, and a stable condition if *θ*_−_ ≤ *r*_*d*_ ≤ *θ*_+_.

(II) Amplitude criterion. The ratio *r*_*f*_ of the sum of the amplitudes of the trading days with rising index to the sum of the amplitudes of the total trading days in a specific time window is given by

$$\begin{array}{c} r_{f}=\frac{\sum_{t\in {T_{i}}^{+}}\left|P\left(t\right)-P\left(t-1\right)\right|}{\sum_{t\in T_{i}}\left|P\left(t\right)-P\left(t-1\right)\right|}, \end{array}$$ (5)

where ${T_{i}}^{+}$ is the set of days in which the closing price is larger than that of the previous day, *T*_*i*_ is the set of all the trading days in the *i*-th time window, and *P*(*t*) is the closing price on the *t*-th day. Similarly, with the thresholds *θ*_+_ and *θ*_−_, we identify a drawup condition if *r*_*f*_ > *θ*_+_, a drawdown condition if *r*_*f*_ < *θ*_−_, and a stable condition if *θ*_−_ ≤ *r*_*f*_ ≤ *θ*_+_.

(III) “OR” criterion. We identify a drawup condition if *r*_*d*_ > *θ*_+_ or *r*_*f*_ > *θ*_+_, and a drawdown condition if *r*_*d*_ < *θ*_−_ or *r*_*f*_ < *θ*_−_. A stable condition is identified if *θ*_−_ ≤ *r*_*d*_ ≤ *θ*_+_ and *θ*_−_ ≤ *r*_*f*_ ≤ *θ*_+_. Situations like *r*_*d*_ > *θ*_+_ and *r*_*f*_ < *θ*_−_, or *r*_*f*_ > *θ*_+_ and *r*_*d*_ < *θ*_−_ do not exist for the thresholds that we choose.

(IV) “AND” criterion. We identify a drawup condition if *r*_*d*_ > *θ*_+_ and *r*_*f*_ > *θ*_+_, and a drawdown condition if *r*_*d*_ < *θ*_−_ and *r*_*f*_ < *θ*_−_. A stable condition is identified if *θ*_−_ ≤ *r*_*d*_ ≤ *θ*_+_ or *θ*_−_ ≤ *r*_*f*_ ≤ *θ*_+_. Situations like *r*_*d*_ > *θ*_+_ and *r*_*f*_ < *θ*_−_, or *r*_*f*_ > *θ*_+_ and *r*_*d*_ < *θ*_−_ do not exist for the thresholds that we choose.

**Fig 1:**
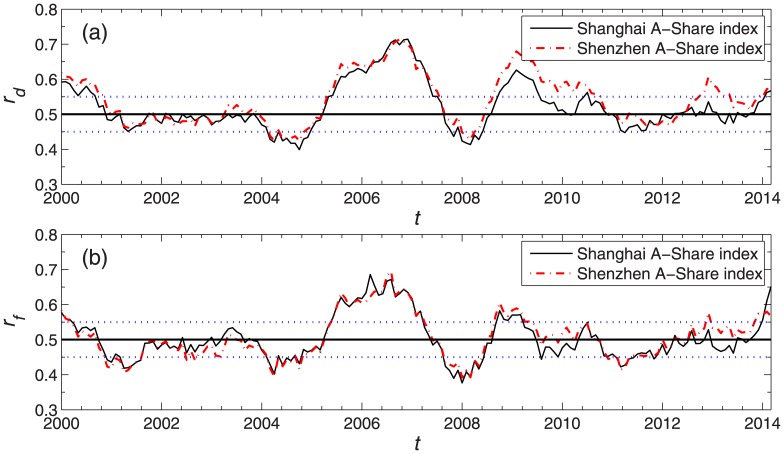
Ratios *r*_*d*_ and *r*_*f*_. Ratios *r*_*d*_ and *r*_*f*_ as a function of time *t*, together with the thresholds *θ*_+_ = 0.55 and *θ*_−_ = 0.45 depicted by blue dotted lines.

The ratios *r*_*d*_ and *r*_*f*_ over time are shown in Fig 1, where each point on the curve is measured by using the market indices in a 10-month horizon following this point. In the figure, the patterns of *r*_*d*_ and *r*_*f*_ show some differences, which cause slight distinctions in the identification of market conditions. In our study, we choose *θ*_+_ = 0.55, *θ*_−_ = 0.45 as the thresholds. Theoretically, other choices of thresholds will also work in our study. For a larger value of *θ*_+_ and a smaller value of *θ*_−_, the number of samples of drawup or drawdown conditions is not statistically sufficient for the lack of data. For a smaller value of *θ*_+_ and a larger value of *θ*_−_, the drawup and drawdown conditions cannot be distinctly identified. Other suitable choices of thresholds around *θ*_+_ = 0.55, *θ*_−_ = 0.45 have also been studied and the results do not change significantly.

**Fig 2:**
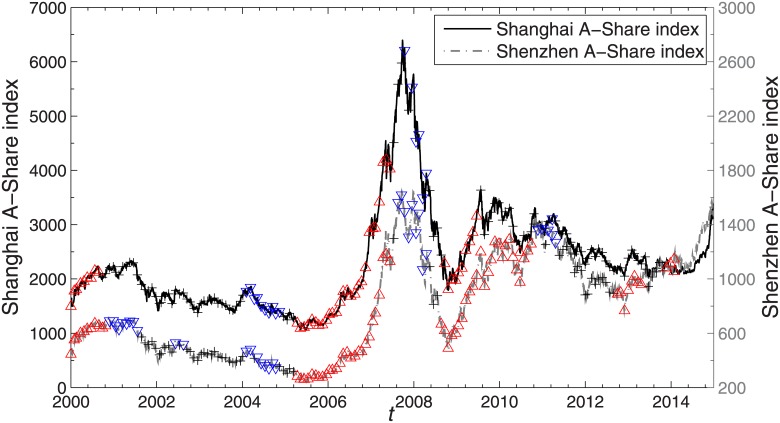
Illustration of market condition. Market conditions identified based on trading day criterion for the Shanghai and Shenzhen A-Share markets. Market conditions include drawup (upright triangle), drawdown (inverted triangle) and stable (cross) conditions.

Market conditions identified based on the trading day criterion for the Shanghai and Shenzhen A-Share markets are shown in Fig 2. The upright triangle indicates a drawup horizon from the current time to 10 months later. Similarly the inverted triangle indicates a drawdown horizon and the cross symbol indicates a stable horizon. It appears that most of the drawup and drawdown conditions identified in our study are proper and sustainable in both markets.

For each time window we get three possible market conditions. We thus get nine combinations of market conditions in the selection horizon and the following investment horizon: drawup in both selection horizon and investment horizon (UU), drawup in the selection horizon and stable in the investment horizon (US), drawup in the selection horizon and drawdown in the investment horizon (UD), stable in the selection horizon and drawup in the investment horizon (SU), stable in both selection and investment horizons (SS), stable in the selection horizon and drawdown in the investment horizon (SD), drawdown in the selection horizon and drawup in the investment horizon (DU), drawdown in the selection horizon and stable trend in the investment horizon (DS), and drawdown in both selection and investment horizons (DD).

## Results

### Evolution of network structures

**Fig 3:**
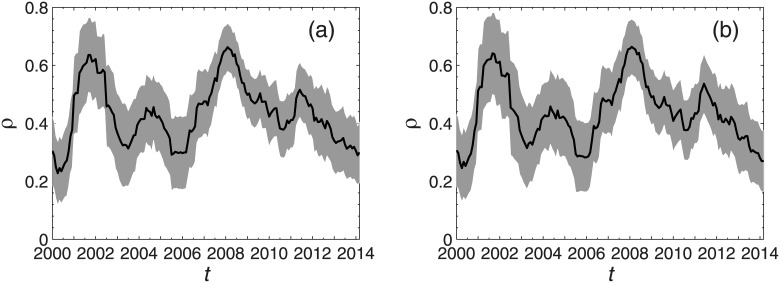
Evolution of average correlation coefficient. Evolution of the average of the correlation coefficients in the Shanghai A-Share market (a) and Shenzhen A-Share market (b). The average of the correlation coefficients are shown by the black solid lines in the center, and correlation coefficients ranging within one standard deviation of the average are shown in the grey area.

Practically speaking, the network structure is evolving over time and changes remarkably during crises. Some evolutionary characteristics of the market can be found from the topological parameters of the networks. First, the average of the correlation coefficients among all stocks reflect the overall connections of the spanning tree, which is shown in Fig 3. To learn more about the details of the evolution of correlation coefficients in each window, the correlation coefficients ranging within one standard deviation of the average value are also shown in the figure. The average of the correlation coefficients rises sharply during the periods of market crashes in 2001 and 2008. As the market recovers, the average of the correlation coefficients declines correspondingly. This finding is consistent with the conclusions illustrated by previous research works, indicating that the connection between stocks will be enhanced in a crisis [10, 34].

**Fig 4:**
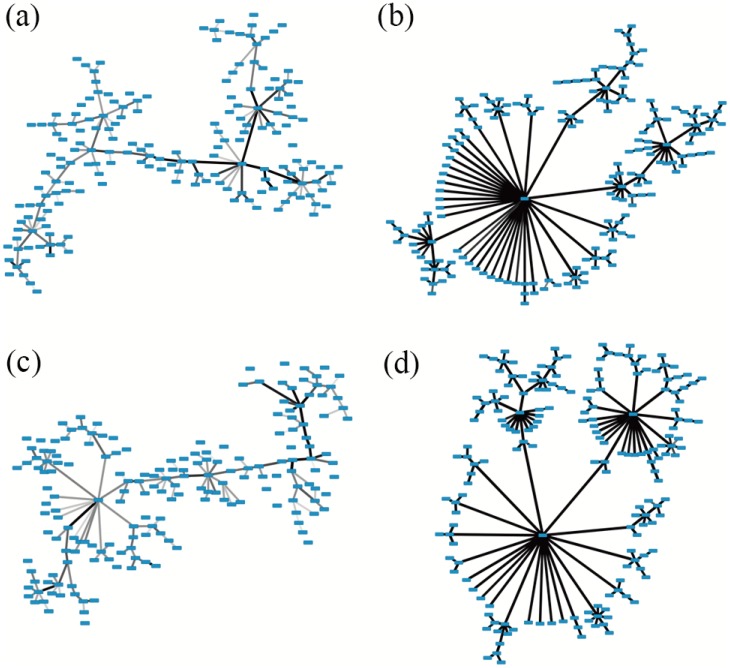
Evolution of network structure. (a) MST network for the Shanghai A-Share market in the period from January 1, 2000 to October 31, 2000; (b) MST network for the Shanghai A-Share market in the period from January 1, 2008 to October 31, 2008; (c) MST network for the Shenzhen A-Share market in the period from January 1, 2000 to October 31, 2000; (d) MST network for the Shenzhen A-Share market in the period from January 1, 2008 to October 31, 2008. Distances between stocks are indicated by line width: A thicker line represents a shorter distance while a thinner line represents a longer distance.

To better understand the variance of the network structure over time *t*, we choose two typical examples of MST networks beginning at January 1, 2000 and January 1, 2008, when stock prices are stable in the former window but volatile in the latter. Their network structures are shown in Fig 4. We can see that the distances between stocks are much smaller, and the corresponding network shrinks to a large extent during the 2008 market crisis.

Other parameters, namely degree, betweenness centrality, distance on degree criterion, distance on correlation criterion and distance on distance criterion, are more specific than the correlation coefficient, can therefore tell us more information about the stocks and networks. These parameters will be used for selecting portfolios in our later study.

**Fig 5:**
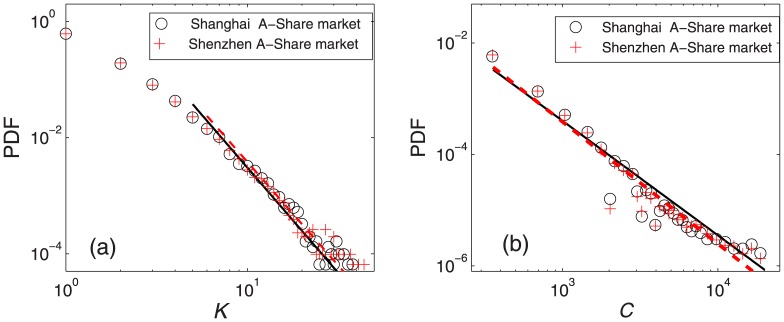
PDFs of *K* and *C*. PDF of degree *K* and betweenness centrality *C* in the Shanghai A-Share market (a) and the Shenzhen A-Share market (b). The fitted lines with exponents estimated by a maximum likelihood estimation method proposed by Newman are for the Shanghai A-Share (black solid line) and Shenzhen A-Share markets (red dashed line).

The parameter degree *K* describes the relationship between a stock and its neighbors. Many studies have found that the degree in empirical networks follows a power-law distribution [48, 53, 58], where the distribution of *K* takes the form *P*(*K*) ∼ *K*^−*α*^. Here, we observe a power-law behavior for the probability density function (PDF) of *K* in Fig 5. A maximum likelihood estimation method proposed by Newman is used to fit the distributions [59]. The exponents *α* = 3.6202 and 3.5597 are obtained for *P*(*K*) in the Shanghai and Shenzhen A-Share markets respectively. Another parameter, the betweenness centrality *C* reflects the contribution of a stock to the connectivity of the whole network, resembling the degree to some extent. The PDF of *C*, also shown in Fig 5, shows a power-law behavior. The exponents *α* = 2.0562, and 2.1927 are estimated for *P*(*C*) in the Shanghai and Shenzhen A-Share markets respectively. Results in the figure show the scale free property of MST networks in the two markets, and also the intense connection between stocks existing in a small number of central stocks, the volatility of which might have a great impact on its neighbors.

**Fig 6:**
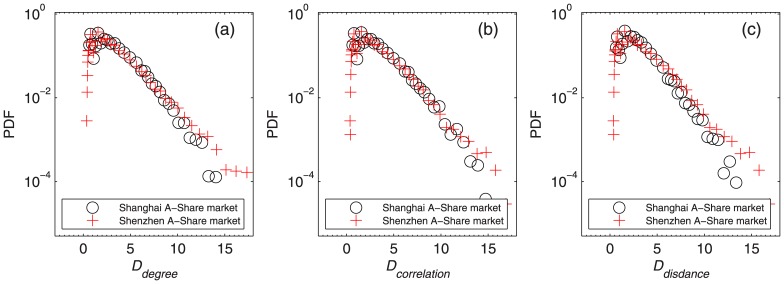
PDF of distance. (a) PDF of distance based on degree criterion *D*_*degree*_, (b) PDF of distance based on correlation criterion *D*_*correlation*_ and (c) PDF of distance based on distance criterion *D*_*distance*_ in the Shanghai A-Share and Shenzhen A-Share markets.

Distance, which is the total length from a node to the central node of the network, has three categories according to the choice of central node in our study, namely distance on degree criterion *D*_*degree*_, distance on correlation criterion *D*_*correlation*_ and distance on distance criterion *D*_*distance*_. Their PDFs are shown in Fig 6. Although the three categories of distances differ from each other, their distributions as well as the stocks selected by these criteria share similar behavior. As can be seen from Fig 6, few stocks are very distant from the central node, while most stocks are at a relatively short distance from the central node. In addition, we note that the MST networks change over time, its central node therefore also changes.

### Comparison between portfolio strategies under different market conditions

In this subsection, we compare the returns of the central and peripheral portfolios to look for the optimal portfolio among these portfolios. The portfolios are selected by using five parameters, i.e., *K*, *C*, *D*_*degree*_, *D*_*correlation*_ and *D*_*correlation*_ in the selection horizon, and the returns of the selected portfolios are calculated in the following investment horizon. The length of the investment horizon is set to be 10 months as discussed in Determination of investment horizon in Methods. In this paper, the selection horizon lasts from January 1, 2000 to February 31, 2014, and the investment horizon lasts from November 1, 2000 to December 31, 2014. We thus have a total of 161 daily points to be used for portfolio investments.

We calculate the returns of the central and peripheral portfolios, and use the returns of random portfolios as a benchmark. A random portfolio is defined as a randomly selected portfolio containing 10% of the total stocks. We first classify the samples of returns of selected portfolios and random portfolios into groups according to the nine combinations of market conditions identified using thresholds *θ*_+_ = 0.55, *θ*_−_ = 0.45 based on the trading day criterion. For each combination of market conditions, we calculate the average return of each stock in the group of selected portfolios and the average return of each stock in the group of random portfolios. The difference between the average returns of selected portfolios and random portfolios is defined as the excess return. Furthermore, a one-way ANOVA is used to test the equality of the excess returns between central portfolios and peripheral portfolios under the same market condition. Null hypothesis of the one-way ANOVA, in which the excess returns of central portfolios and peripheral portfolios are equal, are tested at a specific significance level. If the null hypothesis is rejected, the excess returns of the two portfolios are significantly different. If the null hypothesis cannot be rejected, there is no significant difference between the excess returns of the central portfolios and the peripheral portfolios.

The results of the one-way ANOVA test and the excess returns of the central and the peripheral portfolios for the Shanghai and Shenzhen A-Share markets are listed in Tables 3 and 4 respectively. Notice that if the sample number is less than 11 under a specific combination of market conditions, we do not show the result of this combination for the lack of data. In addition, if there is no significant difference between the excess returns of the central portfolios and the peripheral portfolios, the results are also not shown. In Table 3, we can see from the *p*-value that the excess returns between the central and peripheral portfolios of the listed groups are all significantly different at the 10% level, and most of them are significantly different at the 5% level. By comparing the excess returns of the central and the peripheral portfolios under all listed combinations of market conditions, we find that the central portfolios are more profitable except for two cases. More specifically, when the market is stable in the investment horizon, and whether the market is stable or drawup in the selection horizon, the excess returns of central portfolios are significantly larger than those of peripheral portfolios. When the market is drawdown in the selection horizon and drawup in the investment horizon, or when the market is stable in the selection horizon and drawup in the investment horizon, the central portfolios outperform the peripheral portfolios when using *K*, *D*_*degree*_, *D*_*correlation*_ and *D*_*distance*_ as parameters. Besides, when the market is stable in the selection horizon and drawdown in the investment horizon, the peripheral portfolios gain more than the central portfolios when using *D*_*degree*_ and *D*_*distance*_ as parameters. However, under the same market condition and using *K* as the parameter, the optimal portfolio is the central portfolio with a relatively small gap between the excess returns of the two portfolios. In Table 4 for the Shenzhen A-Share market, we find that the results generally coincide with those for the Shanghai A-Share market. The central portfolios outperform the peripheral portfolios under every combination of market conditions when there exist significant difference between their returns. What is more, the central portfolios selected by using *K*, *D*_*degree*_, *D*_*correlation*_ and *D*_*distance*_ outperform the peripheral portfolios when the market goes through a rising trend in both selection and investment horizons.

**Table 3:** Comparison between excess returns of central and peripheral portfolios for Shanghai A-Share market.

Parameter	Market condition	Num	*f*-value	*p*-value	excess returns	
					central	peripheral
*K*	US	24	15.17	0.00**	**0.04±0.01**	0.00±0.00
	SS	74	6.75	0.01**	**0.02±0.01**	0.00±0.00
	SD	11	5.79	0.03**	**0.02±0.02**	0.00±0.00
	DU	11	3.87	0.06*	**0.03±0.02**	0.00±0.00
*D*_*degree*_	US	24	5.69	0.02**	**0.02±0.01**	-0.03±0.02
	SU	12	4.27	0.05*	**0.02±0.02**	-0.04±0.02
	SS	74	21.39	0.00**	**0.02±0.01**	-0.03±0.01
	SD	11	3.57	0.07*	-0.02±0.02	**0.04±0.02**
	DU	11	53.08	0.00**	**0.05±0.02**	-0.08±0.02
*D*_*correlation*_	US	24	5.33	0.03**	**0.02±0.01**	-0.03±0.02
	SU	12	4.27	0.05*	**0.02±0.02**	-0.04±0.02
	SS	74	20.82	0.00**	**0.02±0.01**	-0.03±0.01
	DU	11	52.77	0.00**	**0.05±0.02**	-0.08±0.02
*D*_*distance*_	US	24	3.98	0.05*	**0.01±0.01**	-0.03±0.01
	SU	12	4.18	0.05*	**0.01±0.02**	-0.03±0.02
	SS	74	19.81	0.00**	**0.03±0.01**	-0.03±0.01
	SD	11	4.36	0.05*	-0.02±0.02	**0.04±0.02**
	DU	11	56.84	0.00**	**0.05±0.02**	-0.08±0.02

Excess returns of central and peripheral portfolios in the Shanghai A-Share market are compared based on one-way ANOVA. Excess returns of central and peripheral portfolios are selected with respect to five parameters, i.e., *K*, *C*, *D*_*degree*_, *D*_*correlation*_ and *D*_*correlation*_, under different combinations of market conditions based on trading day criterion. The listed variables include the number of samples (Num), *f*-value and *p*-value of one-way ANOVA, excess returns of central and peripheral portfolios under each combination of market conditions. Results which are not significant or calculated with less than 11 samples are not shown. (*indicates significance at 10% level, **indicates significance at 5% level. Figures after ± indicate standard error).

**Table 4:** Comparison between excess returns of central and peripheral portfolios for Shenzhen A-Share market.

Parameter	Market condition	Num	*f*-value	*p*-value	excess returns	
					central	peripheral
*K*	UU	37	2.87	0.09*	**0.02±0.01**	0.00±0.00
	US	32	9.75	0.00**	**0.03±0.01**	-0.01±0.00
	SU	20	3.93	0.05*	**0.03±0.01**	0.00±0.00
	SS	50	19.83	0.00**	**0.02±0.01**	-0.01±0.00
*C*	SS	50	8.00	0.01**	**0.01±0.01**	-0.01±0.00
*D*_*degree*_	UU	37	10.35	0.00**	**0.02±0.01**	-0.04±0.01
	SU	20	20.66	0.00**	**0.05±0.01**	-0.05±0.02
	SS	50	8.50	0.00**	**0.02±0.01**	-0.01±0.01
*D*_*correlation*_	UU	37	3.06	0.08*	**0.00±0.01**	-0.03±0.01
	US	32	2.82	0.10*	**0.03±0.01**	-0.01±0.01
	SU	20	20.66	0.00**	**0.05±0.02**	-0.05±0.02
	SS	50	11.91	0.00**	**0.03±0.01**	-0.02±0.01
*D*_*distance*_	UU	37	6.62	0.01**	**0.01±0.01**	-0.03±0.01
	SU	20	4.76	0.04**	**0.02±0.01**	-0.04±0.02
	SS	50	10.63	0.00**	**0.02±0.01**	-0.02±0.01

Excess returns of central and peripheral portfolios in the Shenzhen A-Share market are compared based on one-way ANOVA. Excess returns of central and peripheral portfolios are selected with respect to five parameters, i.e., *K*, *C*, *D*_*degree*_, *D*_*correlation*_ and *D*_*correlation*_, under different combinations of market conditions based on trading day criterion. The listed variables include the number of samples (Num), *f*-value and *p*-value of one-way ANOVA, excess returns of central and peripheral portfolios under each combination of market conditions. Results which are not significant or calculated with less than 11 samples are not shown. Figures after ± indicate standard error. (*indicates significance at 10% level, **indicates significance at 5% level. Figures after ± indicate standard error).

**Fig 7:**
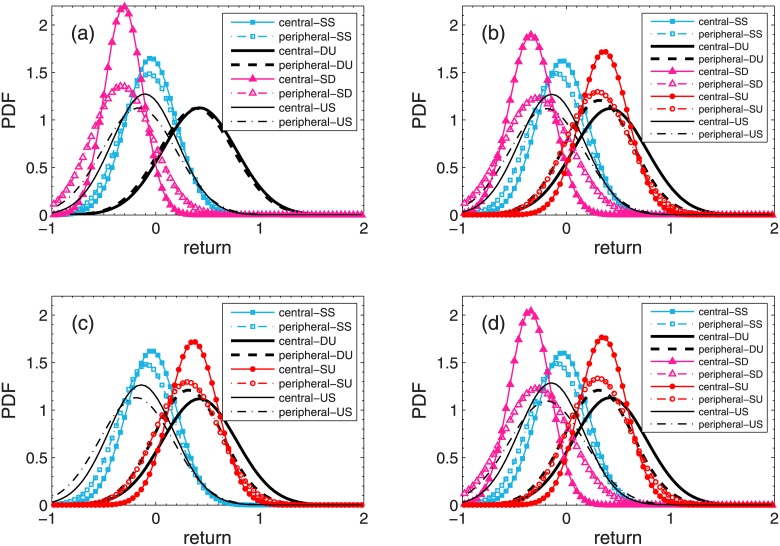
PDF of individual stock returns for Shanghai A-Share Market. PDF of individual stock returns under each combination of market conditions based on trading day criterion in the Shanghai A-Share Market are plotted. Stock portfolios are selected with respect to degree *K* (a), distance based on degree criterion *D*_*degree*_ (b), distance based on correlation criterion *D*_*correlation*_ (c), and distance based on distance criterion *D*_*distance*_ (d).

**Fig 8:**
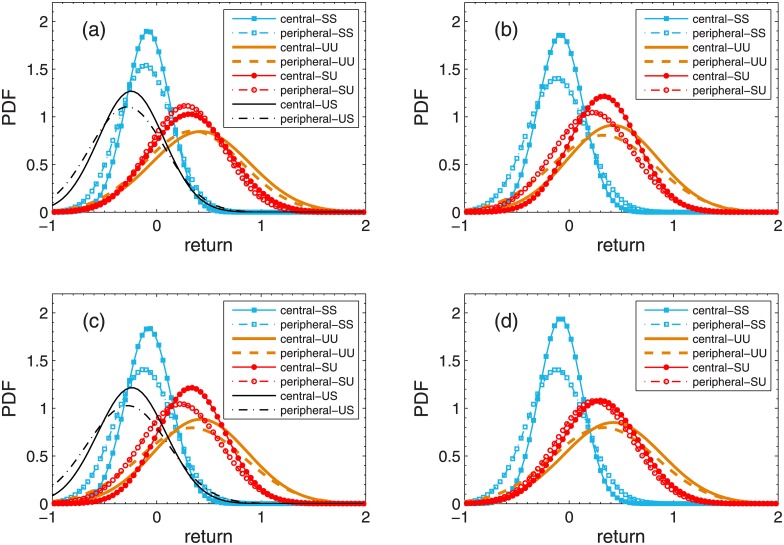
PDF of individual stock returns for Shenzhen A-Share Market. PDF of individual stock returns under each combination of market conditions based on trading day criterion in Shenzhen A-Share Market are plotted. Stock portfolios are selected with respect to degree *K* (a), distance based on degree criterion *D*_*degree*_ (b), distance based on correlation criterion *D*_*correlation*_ (c), and distance based on distance criterion *D*_*distance*_ (d).

We here give the PDF of the returns of individual stocks in the central and peripheral portfolios for the Shanghai and Shenzhen A-Share markets in Figs 7 and 8 respectively. Under most market conditions, the peak of the distribution for the central portfolios in Fig 7 is on the right side of the peak for the peripheral portfolios, indicating that the returns of the central portfolios are on the average larger than those of the peripheral portfolios. However, when portfolios are selected with respect to *D*_*degree*_ or *D*_*distance*_, the peak of the distribution for the peripheral portfolios is on the right side of the peak for the central portfolios under market conditions of SD. These results are consistent with the results from Table 3. We can also see from Fig 7 that the returns of the central portfolios distribute at a relatively narrow range compared with the returns of the peripheral portfolios under most market conditions, showing the close connection among stocks in the central portfolios. Fig 8 shows the PDFs of the returns of individual stocks for the Shenzhen A-Share market. The peak of the distribution for the central portfolios is on the right side of the peak for the peripheral portfolios under all market conditions, indicating that the returns of the central portfolios are on the average larger than those of the peripheral portfolios. Meanwhile, all the returns of the central portfolios have narrow distributions when compared with the returns of the peripheral portfolios, showing the diversification of returns of stocks in the peripheral portfolios.

Similar tests and comparisons are carried out using the amplitude criterion, the “AND” criterion and the “OR” criterion in the identification of market conditions for both markets. Using the amplitude criterion and the “AND” criterion, the results are very similar to those using the trading day criterion. For the “OR” criterion, we find that if the market goes through a turnaround, falling in the selection horizon and rallying in the investment horizon, the central portfolios are proven to be more profitable. More comprehensively, extra efforts have been made for other values of thresholds in the identification of market conditions. Despite some statistical distinctions, the conclusion here shows great similarities with the above, which in turn confirm the reliability of our conclusion.

We summarize and interpret our results in three aspects. First, if the market rises in the investment horizon, the central portfolios should be the best choice. Specifically, if the market has a drawup trend in both the selection and investment horizons, stocks in central portfolios will more likely rise due to their collective movements in rising, while stocks in peripheral portfolios may be too diversified to make profits. If the market rises in the investment horizon after declining, stocks in central portfolios will more likely suffer losses in the selection horizon, and will more likely rise in the investment horizon after hitting rock bottom. Second, if the market is stable in the investment horizon and has just gone through a drawup or stable trend in the selection horizon, central portfolios are preferred. Since stocks in central portfolios are closely related, their prices move in similar behaviors. After the drawup or stable trend in the selection horizon, stocks in central portfolios tend to maintain the drawup or stable trend in the investment horizon. Returns of stocks in peripheral portfolios are too diversified in the stable investment horizon, some negative returns of individual stocks will more likely be in the portfolio. Finally, if the market falls after a period of stable fluctuations, the peripheral portfolios are preferred to avoid risks. The diverse characteristic of the peripheral portfolios is a good way to reduce risk and secure capital.

### Empirical test of optimal portfolio strategy

We have compared the performances of the central and the peripheral portfolios under different combinations of market conditions. We will attempt to choose the optimal portfolio strategy and apply the strategy to make real investment based on an empirical test. We use the data from 2000 to 2010 to select the optimal portfolio under each specific combination of market conditions through a training process, and use the selected optimal strategy according to the current market condition to make investment based on the data from 2010 to 2014. The test is performed specifically as follows.

I. Training to find the optimal portfolio strategy. The training process is carried out by using the methods mentioned in the last section to find the optimal portfolios under specific market conditions. A series of MST networks in the selection horizons are constrcuted to build central and peripheral portfolios, which are then used for investment in the following horizon. Excess returns of the central and peripheral portfolios in the investment horizons are calculated and tested using a one-way ANOVA under each market condition. Portfolios with significantly higher excess returns under different market conditions will comprise the optimal portfolio strategy.

II. Applying the optimal strategy to investment. Before investment, market conditions in the investment horizon need to be predicted. The trend of the stock market is found to be affected by many factors such as policy [60, 61] and economic environment [62, 63]. The effect of these factors is quite evident in the Chinese stock market [64–66], which makes it possible to use these factors to project the market trend. Since we mainly concentrate on building portfolios and strategies other than trend prediction, we identify the market conditions in the investment horizon using the empirical data. In other words, our strategy performs well when the market condition in the investment horizon has a clear trend. Based on the identified combination of market conditions, the optimal portfolio of the optimal strategy chosen in step I is selected and used for further investment. If the combination of market conditions do not appear, investment will not be made. The length of the selection and investment horizons are 10 months, same as in the previous section.

We calculate the excess returns of our strategy, which is the difference between the average return of the stocks in the optimal portfolio strategy and the random strategy. The random strategy comprises random portfolios, defined as portfolios containing 10% of the total stocks that are randomly selected, under different combinations of market conditions. Since the optimal strategy changes as we use five parameters to select portfolios and four criteria to identify market conditions, the excess returns which are shown in Table 5 differ correspondingly. It can be seen from the table that in most cases higher profits can be obtained by our strategies compared with the random strategy. Specifically, 65% of the returns of our strategies are larger than those of the random strategy in the Shanghai A-Share market, and the proportion is 70% in the Shenzhen A-Share market. Furthermore, when using *K* as the parameter to select central or peripheral portfolios, returns of our strategies are always higher than those of the random strategy but the portfolios selected by *C* rarely outperform the random portfolios. Since our strategy based on the “AND” criterion in the Shanghai A-Share market is rarely used, the excess returns under which are mostly negative. The most profitable strategy for the Shanghai A-share market uses *D*_*distance*_ as the parameter to select portfolios and identifies market conditions based on the trading day criterion, which has an excess return of 0.0416. For the Shenzhen A-Share market, the most profitable strategy uses *D*_*correlation*_ as the parameter to select portfolios and identifies market conditions based on the trading day criterion, which has an excess return of 0.0632.

**Table 5:** Excess return of optimal portfolio strategy.

	Shanghai A-Share market				Shenzhen A-Share market			
	trading day criterion	amplitude criterion	“OR” criterion	“AND” criterion	trading day criterion	amplitude criterion	“OR” criterion	“AND” criterion
*K*	**0.0169**	**0.0065**	**0.0072**	**0.0156**	**0.0131**	**0.0113**	**0.0190**	**0.0238**
	**±0.0104**	**±0.0068**	**±0.0045**	**±0.0102**	**±0.0119**	**±0.0088**	**±0.0072**	**±0.0076**
*C*	-0.0016	-0.0003	**0.0129**	-0.0033	-0.0030	-0.0135	-0.0118	-0.0049
	±0.0096	±0.0073	**±0.0072**	±0.0083	±0.0090	±0.0087	±0.0117	±0.0000
*D*_*degree*_	**0.0345**	**0.0025**	**0.0072**	-0.1290	**0.0303**	**0.0381**	-0.0093	**0.0529**
	**±0.0106**	**±0.0176**	**±0.0160**	±0.0000	**±0.0150**	**±0.0102**	±0.0208	**±0.0166**
*D*_*correlation*_	**0.0385**	**0.0025**	**0.0072**	-0.1290	**0.0632**	**0.0451**	**0.0542**	**0.0214**
	**±0.0113**	**±0.0176**	**±0.0160**	±0.0000	**±0.0120**	**±0.0132**	**±0.0195**	**±0.0293**
*D*_*distance*_	**0.0416**	-0.0005	**0.0045**	-0.1290	**0.0054**	-0.0110	**0.0286**	**0.0000**
	**±0.0127**	±0.0153	**±0.0142**	±0.0000	**±0.0103**	±0.0098	**±0.0142**	**±0.0089**

Excess returns listed are gained by the optimal portfolio strategy for the Shanghai and Shenzhen A-Share markets. The optimal strategy is chosen from all possible categories of portfolios selected with respect to five parameters, namely *K*, *C*, *D*_*degree*_, *D*_*correlation*_ and *D*_*distance*_, based on four criteria, namely trading day criterion, amplitude criterion, “OR” criterion and “AND” criterion. Excess returns in boldface are positive which have returns larger than the random strategy. Figures after ± indicate standard error.

**Fig 9:**
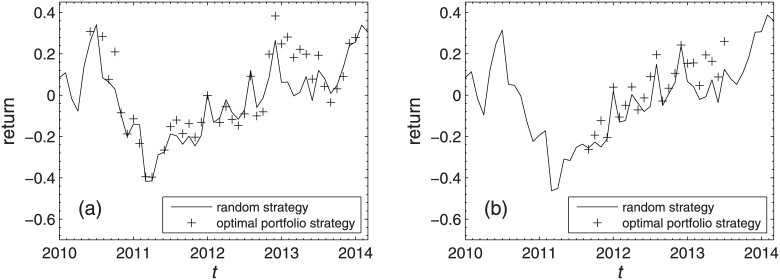
Average return of the most profitable strategy. Average returns of the most profitable strategy (cross) and random strategy (black solid line) for the Shanghai A-Share market (a) and the Shenzhen A-Share market (b). This strategy is described in Table 6.

Table 6 lists the optimal portfolios of the most profitable strategy under different combinations of market conditions for both markets. One can see that the central portfolios are chosen as the optimal portfolios under market conditions of UD, SS and DU, and the peripheral portfolios are chosen under market conditions of SD for the Shanghai A-share market. For the Shenzhen A-Share market, the central portfolios are chosen as the optimal portfolios under market conditions of SU and SS. The average returns of each individual stock in every investment horizon of the most profitable strategy and the random strategy are plotted in Fig 9, where the crosses are the average returns gained by the most profitable strategy in each investment horizon and the black solid line shows the average returns gained by the random strategy. In 65.85% of the investment horizons, the average returns gained by the most profitable strategy are larger than those gained by the random strategy in the Shanghai A-Share market, and the proportion is 91.30% in the Shenzhen A-Share market.

**Table 6:** Optimal portfolios of the most profitable strategy.

Market condition	Shanghai A-Share market				Shenzhen A-Share market	
	UD	SS	SD	DU	SU	SS
Optimal portfolio	central	central	peripheral	central	central	central

Specific optimal portfolios under particular combinations of market conditions comprise the strategy which has the highest returns. The most profitable strategy for the Shanghai A-Share market uses *D*_*distance*_ to select portfolios and identifies market conditions based on trading day criterion, and the most profitable strategy for the Shenzhen A-Share market uses *D*_*correlation*_ to select portfolios and identifies market conditions based on trading day criterion.

**Fig 10:**
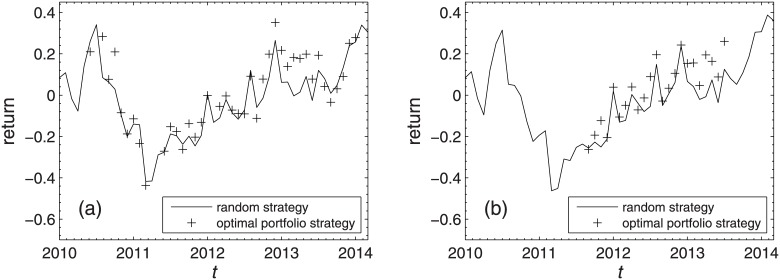
Average return of the strategy with the largest probability of gaining more profits. Average returns of the strategy (cross) that has the largest probability of gaining more profits than the random strategy (black solid line) in the Shanghai A-Share market (a) and the Shenzhen A-Share market (b). This strategy is described in Table 7.

We further find the strategy that has the largest probability of gaining more profits than the random strategy, i.e., it outperforms the random strategy in most of investment horizons. This strategy uses *D*_*correlation*_ as the parameter to select portfolios and identifies market conditions based on the trading day criterion for both markets. The optimal portfolios under different combinations of market conditions are listed in Table 7. One can see that the central portfolios are chosen as the optimal portfolios under market conditions of UD, SS and DU for the Shanghai A-share market. For the Shenzhen A-Share market, this strategy is also the most profitable strategy with the central portfolios chosen as the optimal portfolios under market conditions of SU and SS. Similar to the above, the average returns of each individual stock in every investment horizon of this strategy and the random strategy are plotted in Fig 10. In 70% of the investment horizons, the average returns gained by our strategy are larger than the random strategy in the Shanghai A-Share market, and the proportion is 91.30% in the Shenzhen A-Share market.

**Table 7:** Optimal portfolios of the strategy with the largest probability of gaining more profits.

Market condition	Shanghai A-Share market			Shenzhen A-Share market	
	UD	SS	DU	SU	SS
Optimal portfolio	central	central	central	central	central

Specific optimal portfolios under particular combinations of market conditions comprise the strategy that has the largest probability of gaining more profits than the random strategy. This strategy uses *D*_*correlation*_ as the parameter to select portfolios and identifies market conditions based on trading day criterion for both markets.

## Discussion and conclusion

In this paper, we propose a new dynamic portfolio strategy based on the time-varying structures of MST networks for the Shanghai and Shenzhen A-Share markets. The strategy first select central and peripheral portfolios in the selection horizon using five topological parameters, and uses the selected portfolios for investment in the investment horizon. Nine combinations of market conditions have been considered when comparing the excess returns of the central and the peripheral portfolios, which are identified either by the ratio of the number of trading days with rising index to the total number of trading days, or the ratio of the sum of the amplitudes of the trading days with rising index to the sum of the amplitudes of the total trading days. By picking out the portfolios with larger excess returns under different combinations of market conditions, the optimal portfolios under specific market conditions could be found: (i) If the market is likely to have a drawup trend in the following investment horizon, central portfolios should be the best choice, while the peripheral portfolios usually perform worse for excessive diversification. (ii) If the market is in a relatively stable state in the investment horizon, central portfolios are preferred unless the market just went through a drawdown trend in the selection horizon. (iii) If the market is likely to have a drawdown trend in the investment horizon and the market is stable in the selection horizon, peripheral portfolios should be chosen to reduce risks.

Empirical tests have also been carried out and verified the efficiency of our optimal portfolio strategy. We have used the data from 2000 to 2010 to select the optimal portfolio under each specific combination of market conditions through a training process. The selected optimal strategy is selected according to the current market condition to make investment based on the data from 2010 to 2014. By calculating the excess returns of the optimal portfolio strategies, our strategies are found to outperform the random strategy in most cases. Among all possible optimal portfolio strategies based on different parameters to select portfolios and different criteria to identify market conditions, 65% of our optimal portfolio strategies outperform the random strategy for the Shanghai A-Share market and 70% for the Shenzhen A-Share market. Using degree *K* as the parameter to select central or peripheral portfolios, returns of our strategies are always higher than those of the random strategy. The excess returns of the most profitable strategies in the Shanghai and Shenzhen A-Share markets are 0.0416 and 0.0632 respectively. The strategy that has the largest probability of gaining more profits outperforms the random strategy in 70% of the investment horizons for the Shanghai A-Share market, and 91.30% for the Shenzhen A-Share market.

## Supporting Information

S1 File Data sources. The data sources and the specific URLs and steps to download the raw data used in the paper. A minimal data set is available on http://pan.baidu.com/s/1kUXfj3D.
(PDF)
