# Supplementary material for: Dynamic Portfolio Strategy Using Clustering Approach
Source: PLoS One. 2017 Jan 27;12(1):e0169299. doi: 10.1371/journal.pone.0169299 (PMC5271336; doi:10.1371/journal.pone.0169299)

Procedures for Obtaining Data Used in Dynamic Portfolio Strategy Using Clustering Approach

Stock trading data from RESSET Database

Logging in to RESSET system.

Navigate to the RESSET Portal (http://www2.resset.cn/product/index.jsp?lang=en)

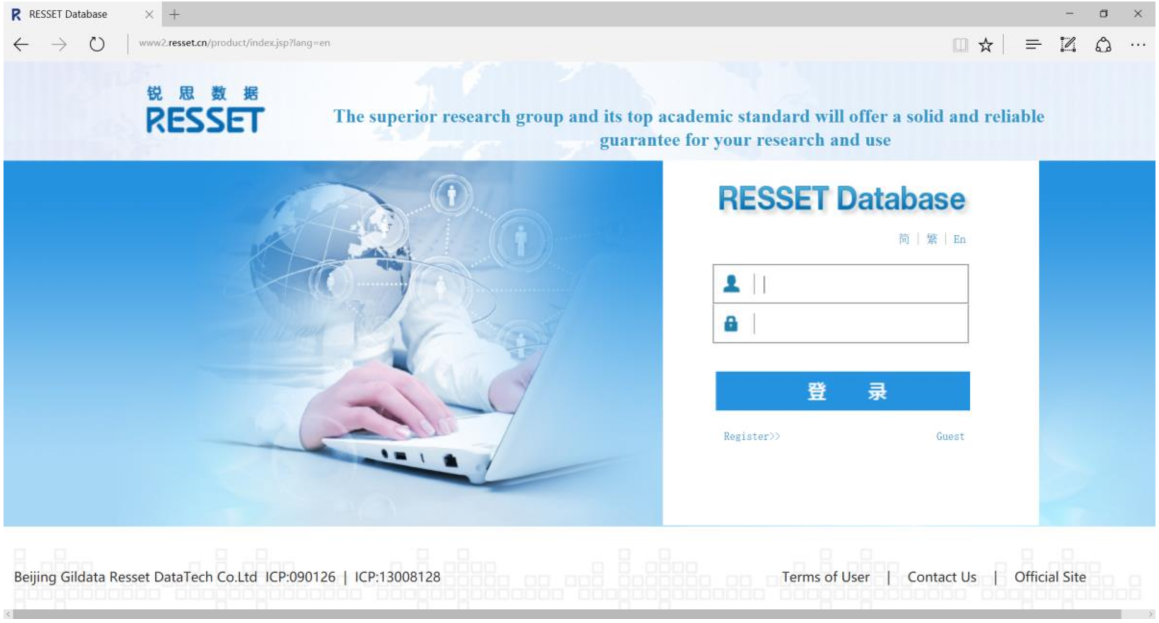

You will see the home page of RESSET Database.

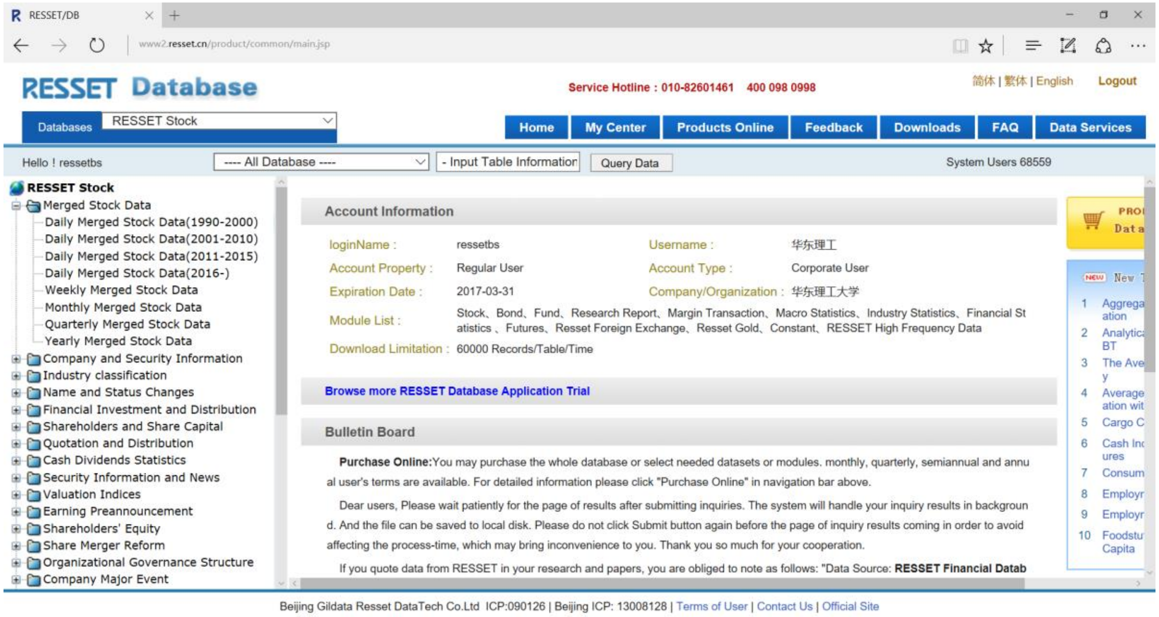

On the left side tab, mouse over ‘Merged Stock Data’, select ‘Daily Merged Stock Data (1990-2000)’, ‘Daily Merged Stock Data (2001-2010)’ or ‘Daily Merged Stock Data (2011-2015)’ to access data in different periods.

On the right side tab, click ‘Code List’ in ‘Step 2 Search Criteria’ and open a new page to select the stocks you want.

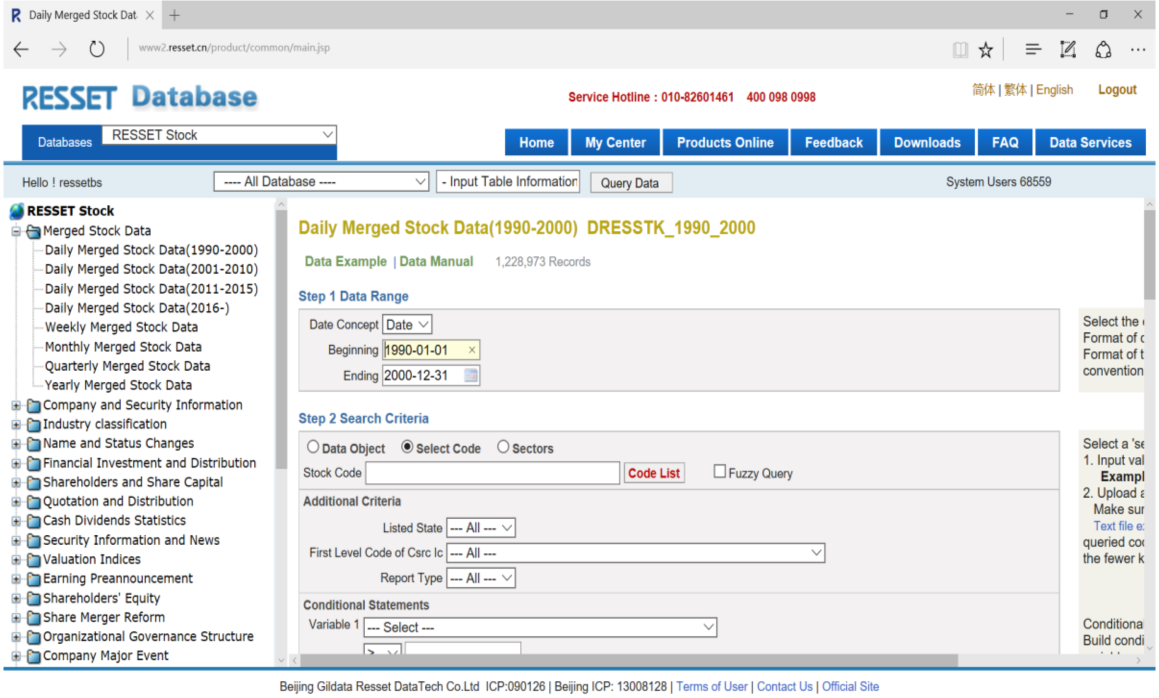

Select '1-Shanghai Stock' or '2-Shenzhen Stock' in 'Exchange Flag' to access stocks listed in Shanghai or Shenzhen stock exchange.

Let the remaining options have the default value and press 'Search'. After choosing stocks from the 'Search Result', click on the 'Confirm' button and back to the former page.

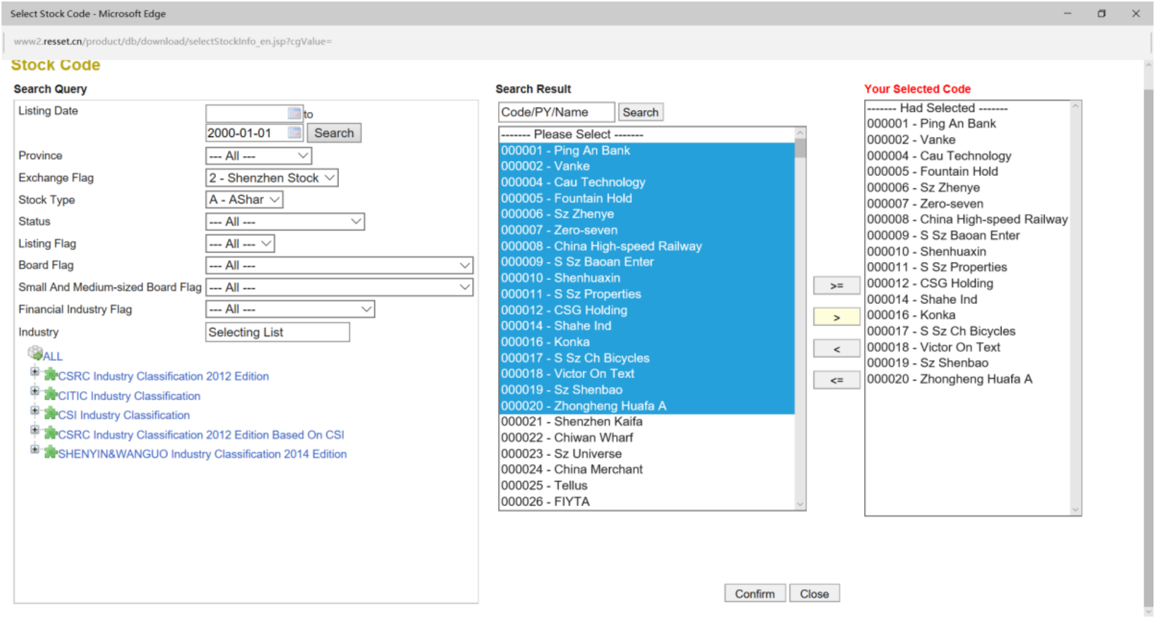

Scroll to the bottom of the page and click on the "Submit" button. Note that the maximum number of records that will be displayed per search differs for different types of users. For us, the maximum number is 60000.

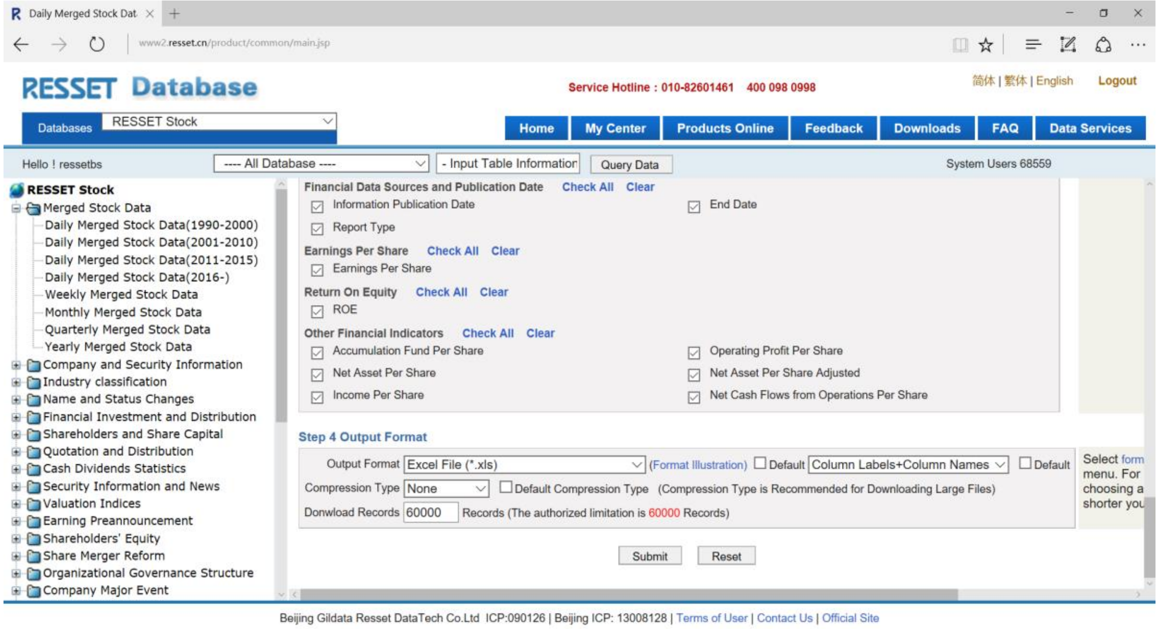

Click on the “Download” button to download the data.

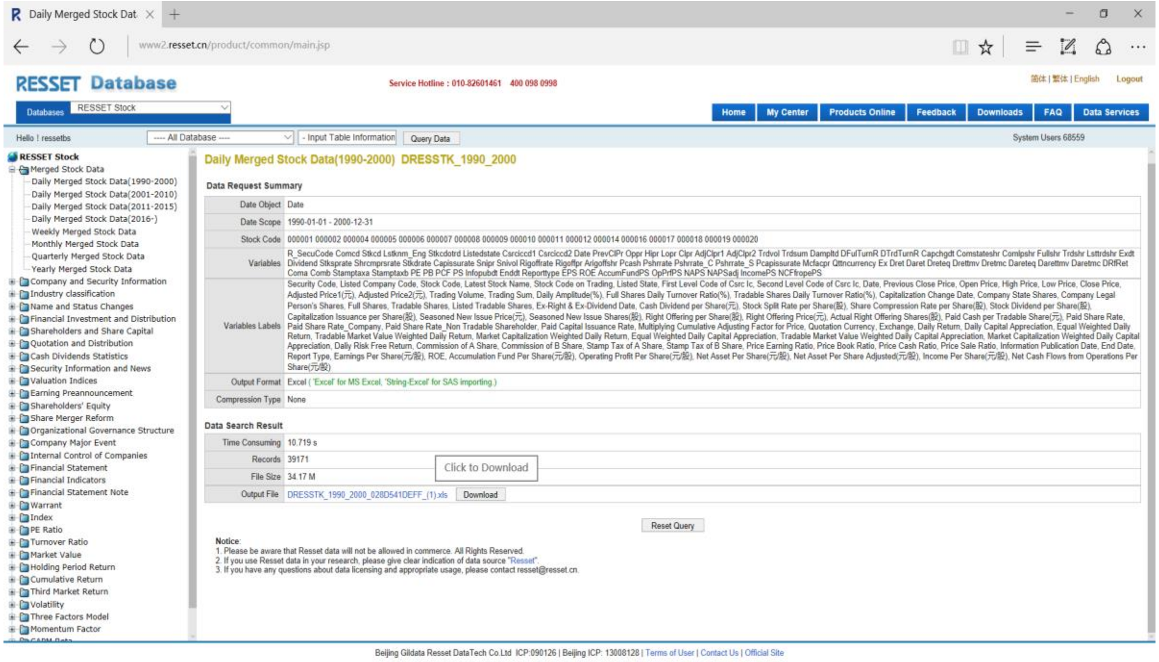

Supplement: S1 File — The data sources and the specific URLs and steps to download the raw data used in the paper. A minimal data set is available on http://pan.baidu.com/s/1kUXfj3D. (PDF) [file pone.0169299.s001.pdf]
